# In Vitro Estrogenic and Breast Cancer Inhibitory Activities of Chemical Constituents Isolated from *Rheum undulatum* L.

**DOI:** 10.3390/molecules23051215

**Published:** 2018-05-18

**Authors:** Dahae Lee, SeonJu Park, Sungyoul Choi, Seung Hyun Kim, Ki Sung Kang

**Affiliations:** 1 School of Pharmacy, Sungkyunkwan University, Suwon 16419, Korea; pjsldh@naver.com; 2 College of Pharmacy, Yonsei Institute of Pharmaceutical Sciences, Yonsei University, Incheon 21983, Korea; seonju88@yonsei.ac.kr; 3 College of Korean Medicine, Gachon University, Seongnam 13120, Korea; pc1075@gachon.ac.kr

**Keywords:** *Rheum undulatum* L., menopause, estrogen receptor, hormone replacement therapy

## Abstract

We investigated the estrogenic and breast cancer inhibitory activities of chemical constituents isolated from Rhei undulati Rhizoma (roots of *Rheum undulatum* L.), which is used as a laxative, an anti-inflammatory, and an anti-blood stagnation agent. Estrogen-like activity was studied using the well characterized E-screen assay in estrogen receptor (ER)-positive MCF-7 cells. The mechanism underlying the breast cancer inhibitory activity of the compounds was studied using human ER-negative MDA-MB-231 and ER-positive MCF-7 cells. The activation of apoptosis pathway-related proteins was investigated by western blotting, using extracts of *R. undulatum* prepared in three solvent conditions (EX1, EX2, and EX3). The *R. undulatum* chemical constituents (compounds **1**–**3**) showed estrogen-like activity in the concentration range of 10 to 50 μM, by increasing the proliferation of human ER-positive MCF-7 cells. These effects were attenuated by co-treatment with 100 nM fulvestrant, an ER antagonist. Compounds **1**–**3** decreased the viability of MCF-7 cells in a concentration-dependent manner. Compounds **1** (aloe emodin) and **2** (rhapontigenin) induced mitochondria-independent apoptosis by activating the caspase-8 pathway, whereas the cytotoxic effect of compound **3** (chrysophanol 1-*O*-β-d-glucopyranoside) was mediated through the mitochondria-dependent apoptotic pathway.

## 1. Introduction

In most middle-aged women, menopause occurs when the ovaries stop synthesizing estrogen [1,2]. Menopausal women exhibit various symptoms and conditions, including anxiety, hot flashes, sweating, insomnia, vaginal dryness, cardiovascular disease, and bone density reduction [1,3,4]. Hormone replacement therapy (HRT), which comprises the administration of estrogens, sex steroids, and progestogens, is used to treat menopause symptoms in women and improves the quality of life of healthy postmenopausal women; however, it is associated with certain risks, such as the occurrence of estrogen receptor (ER)-positive breast cancer [5]. Therefore, the sustained use of HRT is one of the strong risk factors for breast cancer patients [6,7]. 

Previous in vitro and in vivo studies have shown that phytoestrogens may be involved in the growth or proliferation of ER-positive breast cancer cells [8,9]. Thus, developing an alternative to HRT without increasing the risk of breast cancer is very important for improving the quality of life of postmenopausal women. 

Rhubarb is a perennial herb that belongs to the genus *Rheum* L., and many of its ingredients are known constituents of Western and Chinese medicines used to treat constipation. In traditional and Chinese medicines, rhubarb is used as an antipyretic, antiphlogistic, anticoagulant, and anti-jaundice agent [10]. Rhubarb can be divided into official and unofficial classes, depending on its chemical constituents. Unofficial class rhubarbs, that include *Rheum undulatum* Linné, are considered less purgative than official class rhubarbs because of their relatively low levels of anthraquinone derivatives and the absence of sennosides [11]. Anthraquinones have been reported as the most important constituents in rhubarb, exerting various pharmacological efficacies. Stilbenes are also considered the principal ingredients in chemotaxonomy and are present only in unofficial rhubarbs [12].

*R. undulatum* (Polygonaceae) is a well-known and widely used medicinal plant that has been used as an anti-inflammatory, laxative, and anti-blood stagnation agent in Asian countries and is mainly distributed in Korea [11,13]. The important constituents of *R. undulatum* include anthraquinone derivatives, such as aloe-emodin and chrysophanol, and stilbene derivatives, such as rhaponticin and its aglycone, rhapontigenin [10,12,14]. Previous studies have shown that *R. undulatum* had high estrogenic potency in a recombinant yeast system featuring both a reporter plasmid and human ER-expressing plasmid [15]. In addition, the methanolic extracts of *R. undulatum* and anthraquinones enhanced the proliferation of estrogen-sensitive MCF-7 cells [16]. However, estrogenic chemical metabolites of *R. undulatum* have not been reported.

The present study was performed to evaluate the estrogenic and antitumor effects of chemical constituents from *R. undulatum* on the ER-positive MCF-7 and ER-negative MDA MB 231 breast carcinoma cell lines. 

## 2. Results

### 2.1. Comparison of Estrogenic Activities of R. undulatum Extracts (EX1, EX2, and EX3) in the Absence or Presence of Fulvestrant in MCF-7 Cells

**Figure 1:**
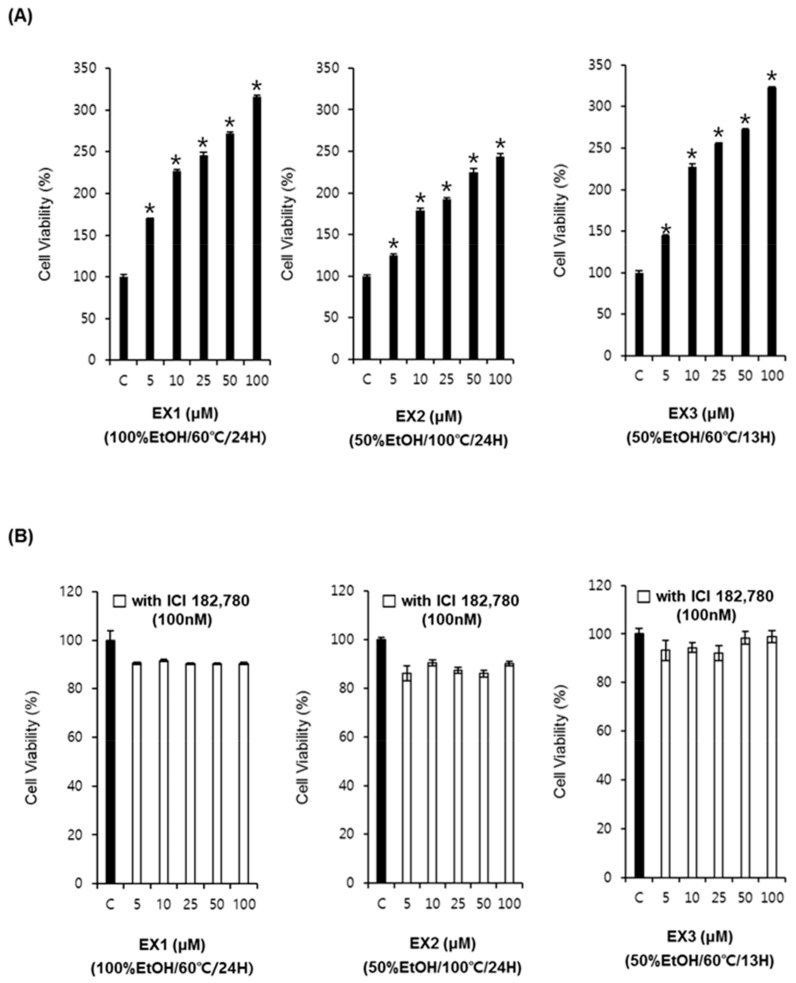
Comparison of the estrogenic activities of *Rheum undulatum* extracts (EX1, EX2, and EX3). (**A**) Comparison of the estrogenic effects of *R. undulatum* extracts on MCF-7 cell proliferation measured by E-screen assay. (**B**) Comparison of the estrogenic effects of *R. undulatum* extracts in the absence or presence of fulvestrant on MCF-7 cell proliferation measured by E-screen assay; * *p* < 0.05 compared to the control value.

We examined the potential estrogenic effect of *R. undulatum* extracts (EX1, EX2, and EX3) on estrogen-responsive MCF-7 cells using an E-screen assay. MCF-7 cells were treated with EX1, EX2, and EX3 at concentrations of 5–100 μg/mL for 6 days. As shown in Figure 1A, EX1, EX2, and EX3 increased the proliferation of MCF-7 cells in a concentration-dependent manner. These effects were attenuated by co-treatment with 100 nM of the ER antagonist fulvestrant (Figure 1B). Thus, the estrogenic activity of the *R. undulatum* extracts was confirmed to occur via the ER. The comparison of efficacy under different extraction conditions revealed excellent efficacy for samples extracted at relatively low temperatures (60 °C) and using a low-polarity solvent (100% EtOH). 

**Figure 2:**
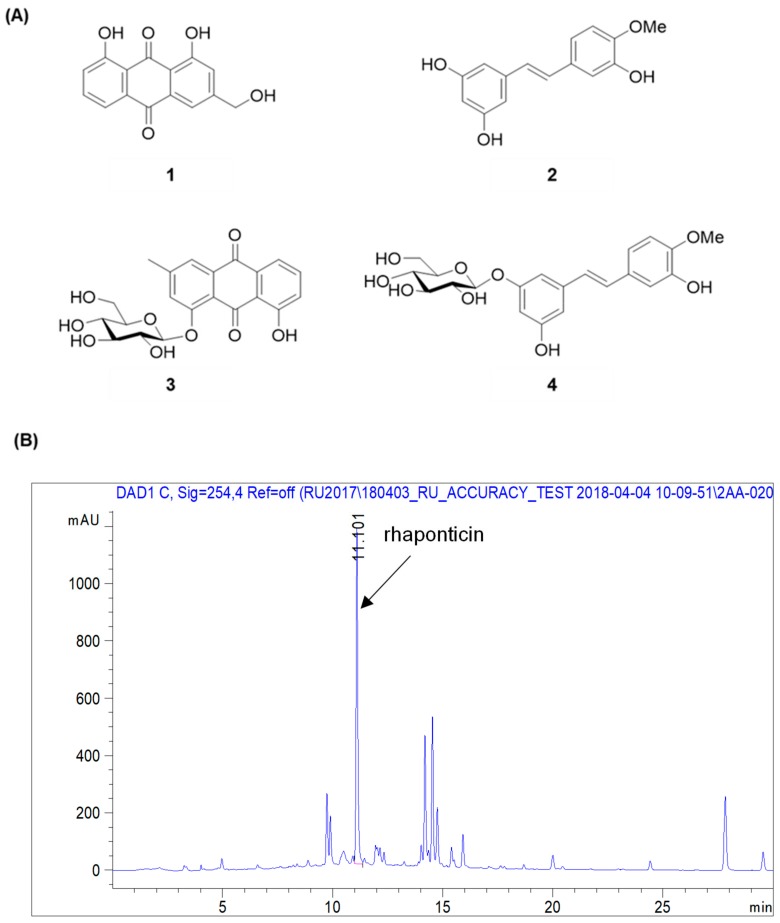
Chemical structures of compounds isolated from an *R. undulatum* extract. (**A**) Chemical structures of four isolated compounds. (**B**) Representative HPLC chromatogram of R. undulatum extract at 254 nm. Compound **1**: emodin, compound **2**: rhapontigenin, compound **3**: chrysophanol **1**-*O*-β-d-glucopyranoside, compound **4**: rhaponticin.

By using spectroscopic methods, including NMR and high-resolution electrospray ionization–mass spectrometry analysis, the compounds isolated from *R. undulatum* (see Materials and Methods) were identified as aloe emodin (compound **1**) [17], rhapontigenin (compound **2**) [18], chrysophanol 1-*O*-β-d-glucopyranoside (compound **3**) [19], and rhaponticin (compound **4**) (Figure 2A) [20]. The contents of rhaponticin in EX1, EX2, and EX3 were 2.98, 14.66, and 15.44 mg/100 mg extracts, respectively, as confirmed by HPLC analysis (Figure 2B). 

### 2.2. Comparison of the Estrogenic Effects of Compounds ***1**–**4*** Isolated from an R. undulatum Extract in the Absence or Presence of Fulvestrant on MCF-7 Cells 

**Figure 3:**
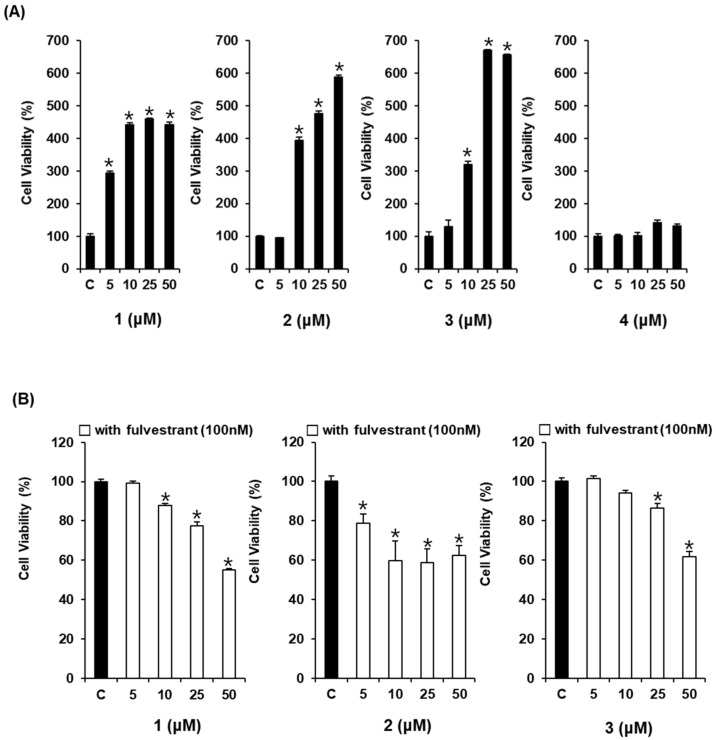
Comparison of the estrogenic effects of compounds **1**–**4** isolated from an *R. undulatum* extract. (**A**) Estrogenic effects of compounds **1**–**4** in the absence of fulvestrant on MCF-7 cell proliferation measured by E-screen assay. (**B**) Estrogenic effects of compounds **1**–**3** in the presence of fulvestrant on MCF-7 cell proliferation measured by E-screen assay. Compound **1**: emodin, compound **2**: rhapontigenin, compound **3**: chrysophanol 1-*O*-β-d-glucopyranoside, compound **4**: rhaponticin; * *p* < 0.05 compared to the control value.

The estrogenic effects of compounds **1**–**4** were evaluated in estrogen-responsive MCF-7 cells using the E-screen assay. This assay was carried out to assess the estrogenicity of chemicals by evaluating the ability of MCF-7 cells to proliferate. To test the effects of compounds **1**–**4** in MCF-7 cells, the cells were treated with these compounds at concentrations of 5–50 μM for 6 days. As shown in Figure 3A, compounds **1**–**3** increased the proliferation of MCF-7 cells in a concentration-dependent manner. The effect of compound **4** was absent or very weak within the error range, so this compound was excluded from the confirmation experiment using the antagonist. These effects were attenuated by co-treatment with ER antagonist fulvestrant at 100 nM (Figure 3B). Therefore, the estrogenic activities of compounds **1**–**3** were mediated by the ER.

### 2.3. Comparison of the Cytotoxic Effects of the Isolated Compounds ***1**–**3*** on the Viability of Human Breast Cancer Cells MDA-MB-231 and MCF-7

**Figure 4:**
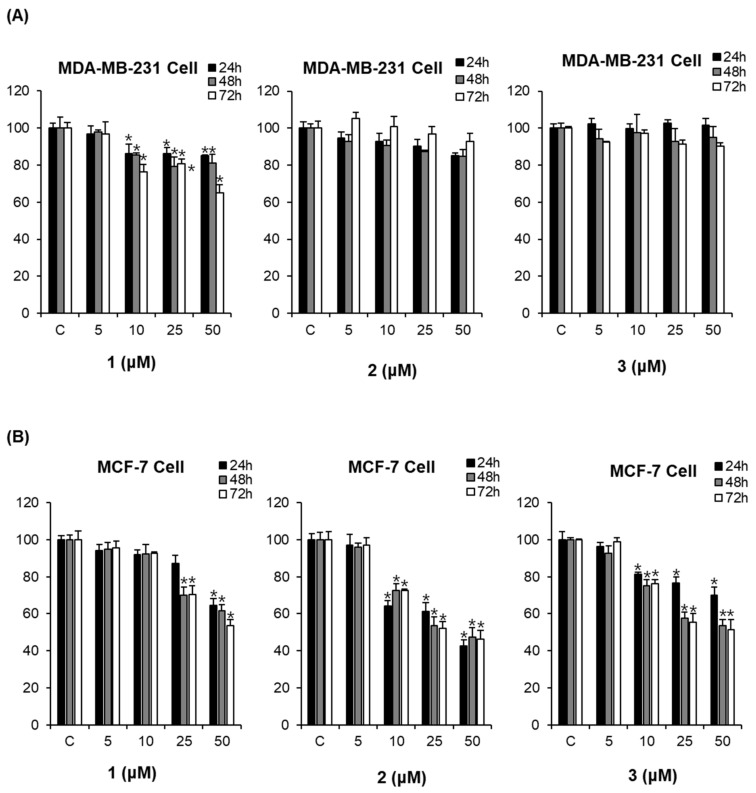
Comparison of the cytotoxic effects of the isolated compounds **1**–**3** on the viability of human breast cancer cells MDA-MB-231 and MCF-7. (**A**) Cytotoxic effects of the isolated compounds on MDA-MB-231 cells. (**B**) Cytotoxic effects of the isolated compounds on MCF-7 cells. Compound **1**: emodin, compound **2**: rhapontigenin, compound **3**: chrysophanol 1-*O*-β-d-glucopyranoside; * *p* < 0.05 compared to the control value.

The cytotoxic effects of compounds **1**–**3** were examined in ER-positive MCF-7 and ER-negative MDA-MB-231 breast cancer cells by using a cell viability assay. The cells were treated with compounds **1**–**3** at concentrations of 5–50 μM for 24 h. All compounds exerted weak cytotoxicity, decreasing ER-negative MDA-MB-231 cell viability by approximately 10% (Figure 4A), which may be because of the difficulty of killing cells that are resistant to chemotherapeutics. As shown in Figure 4B, compounds **1**–**3** decreased the proliferation of MCF-7 cells in a concentration-dependent manner. 

### 2.4. Comparison of the Cytotoxic Effects of Compounds ***1**–**3*** in the Absence or Presence of Fulvestrant on MCF-7 Cells 

**Figure 5:**
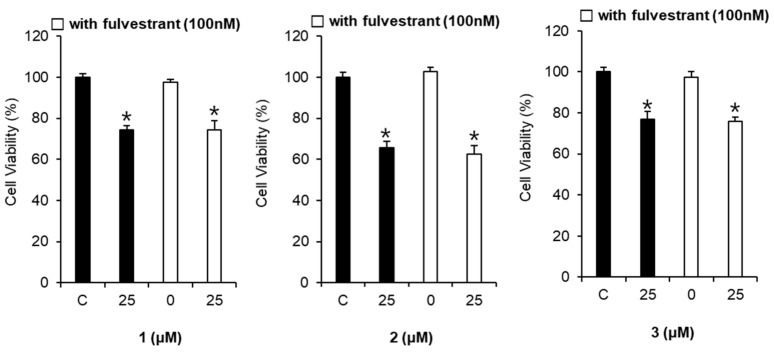
Comparison of the cytotoxic effects of compounds **1**–**3** in the absence or presence of fulvestrant on MCF-7 cell viability. Compound **1**: emodin, compound **2**: rhapontigenin, compound **3**: chrysophanol 1-*O*-β-d-glucopyranoside. * *p* < 0.05 compared to the control value.

The cytotoxic effects of compounds **1**–**3** on MCF-7 cells were unaffected by co-treatment with fulvestrant (100 nM) (Figure 5). These findings revealed that there is no relationship between the cytotoxicity of the compounds and the ER.

### 2.5. Comparison of the Effects of Compounds ***1**–**3*** on the Expression of Various Proteins in MCF-7 Cells

**Figure 6:**
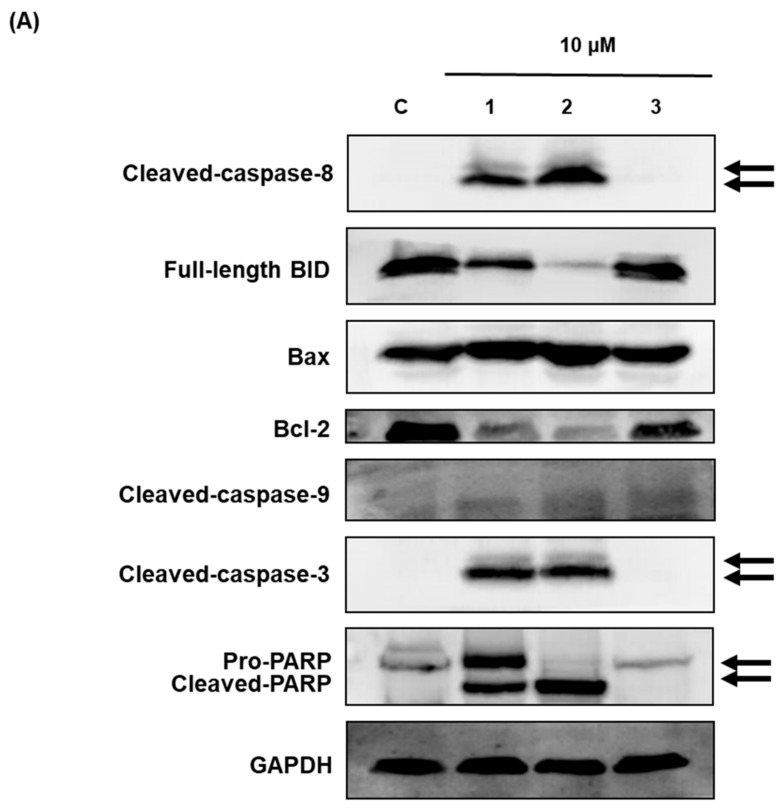

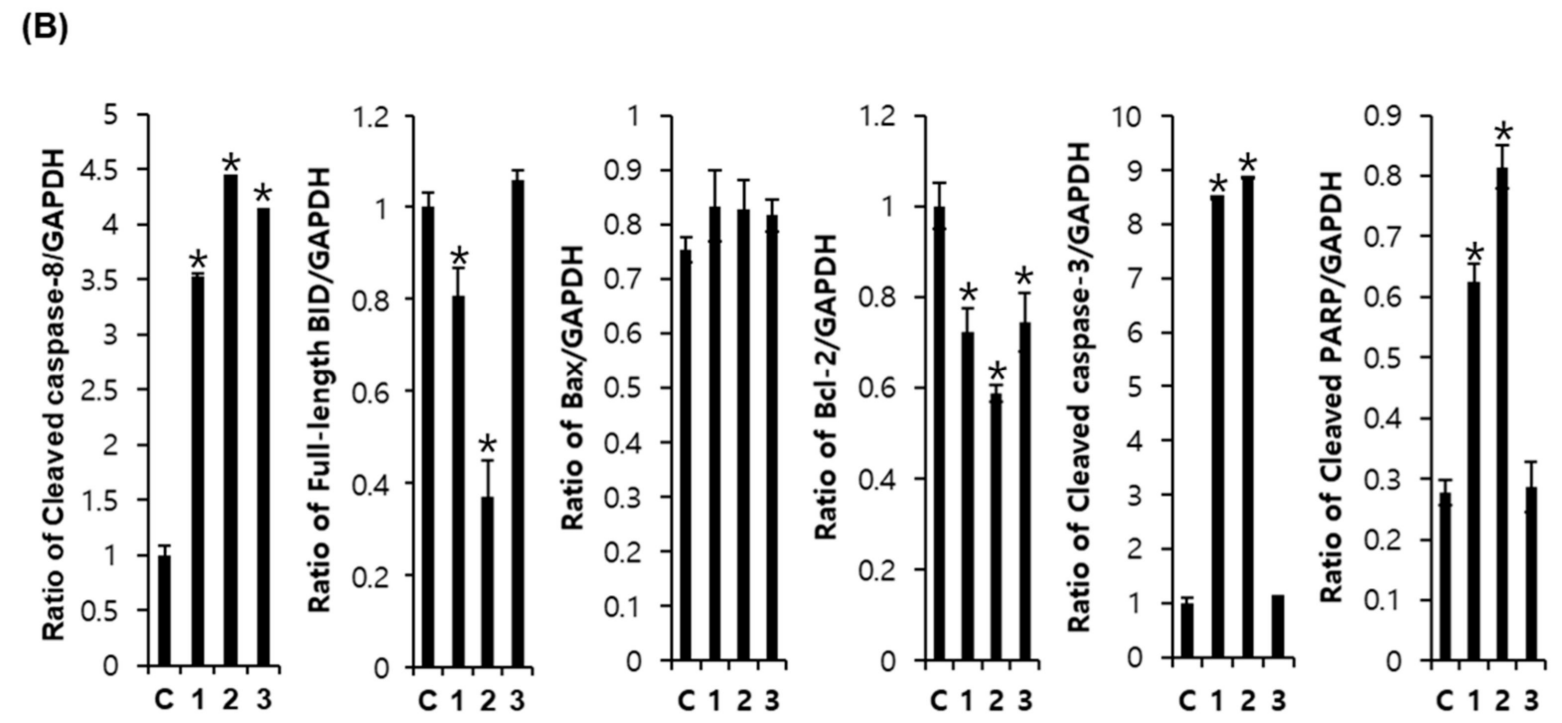
Comparison of the effects of compounds **1**–**3** on the expression of apoptosis-related proteins in MCF-7 cells. (**A**) Expression of cleaved BID, caspase-8, Bax, Bcl-2, cleaved caspase-3, cleaved caspase-9, and PARP in MCF-7 cells. Compound **1**: emodin, compound **2**: rhapontigenin, compound **3**: chrysophanol 1-*O*-β-d-glucopyranoside. (**B**) Each bar graph shows the densitometric quantification of the corresponding western blotting bands. * *p* < 0.05 compared with control value.

Western blot analysis was used to investigate the effects of compounds **1**–**3** on apoptotic cell death pathways in MCF-7 cells. As shown in Figure 6, activation of caspase-3 and caspase-8 and cleavage of PARP was detected after treatment with compounds **1** and **2** in MCF-7 cells. The expression of Bcl-2 and BID was also decreased after treatment with compounds **1** and **2**. However, the active form of caspase-9 and the expression of Bax were not altered by compounds **1**–**3** treatment. These results suggest that compounds **1** (aloe emodin) and **2** (rhapontigenin) induced mitochondria-independent apoptosis by activating the caspase-8 pathway, whereas the cytotoxic effect of compound **3** (chrysophanol 1-*O*-β-d-glucopyranoside) was mediated by mitochondria-dependent apoptosis.

## 3. Discussion

In this study, we investigated the effectiveness of natural products and their ingredients for HRT as well as their side effects such as the increase of breast cancer risk. An E-screen assay was performed to determine whether the isolated compounds have estrogen or anti-estrogen activities in ER-positive MCF-7 cells because these cells proliferate in the presence of estrogens. The proliferative effect was also characterized by examining the inhibitory action of the ER antagonist fulvestrant [21,22,23,24]. *R. undulatum* extracts and three isolated compounds (aloe emodin, rhapontigenin, and chrysophanol 1-*O*-β-d-glucopyranoside) increased the proliferation of MCF-7 cells in a concentration-dependent manner. These effects were attenuated by co-treatment with the anti-estrogen compound fulvestrant, reflecting the estrogenic activity of the *R. undulatum* extract and its ingredients, which were mediated via the ER. Therefore, *R. undulatum* extracts showed estrogenic potency, which is in line with the results of previous studies [25,26]. In addition, we identified new effective compounds (aloe emodin, rhapontigenin, and chrysophanol 1-*O*-β-d-glucopyranoside) in this study.

Breast cancer comprises cells with different types of receptors and thus is affected by hormones. Ten percent of breast cancers are ER/progesterone receptor (PR)-positive and human epidermal growth factor receptor 2 (HER2)-positive, 69% are ER/PR-positive and HER2-negative, 7% are ER/PR-negative and HER2-positive, and the remaining 13% are classified as triple-negative [25,26]. Approximately 50% of breast cancers in postmenopausal women are ER-positive. Estrogens are known to stimulate ER-positive breast cancer cell proliferation. Selective ER-modulators, which bind to the ER and prevent the binding of estrogen, are used to treat ER-positive breast cancer [27,28]. To compare the antitumor and cytotoxic effects of the isolated compounds (aloe emodin, rhapontigenin, and chrysophanol 1-*O*-β-d-glucopyranoside), two different breast cancer cell lines, namely, MCF-7 (low-invasive, ER-negative, estrogen-independent cancer cells) and MDA-MB-231 (highly invasive, ER-positive, estrogen-dependent cancer cells) were used in the present study [7,29,30].

To investigate the side effects, the cytotoxic effects of aloe emodin, rhapontigenin, and chrysophanol 1-*O*-β-d-glucopyranoside on MDA-MB-231 and MCF-7 cells were measured in cell viability assays. These compounds decreased the viability of MCF-7 cells in a concentration-dependent manner. The cytotoxic effects were unaffected by co-treatment with the anti-estrogen compound fulvestrant in MCF-7 cells. Therefore, these cytotoxic effects were independent of the ER.

Previous studies have shown that phytoestrogens function as estrogen antagonists and have anticancer effects in hormone-dependent breast cancer [31,32]. The antitumor effect of soy isoflavones has been reported to be linked to ER modulation, but there is also growing evidence that other pathways are also involved [33]. Among these, the activation of apoptosis, the inhibition of angiogenesis, metastasis, and cell proliferation, or antioxidant effects have been explored using various isoflavones. Several molecular mechanisms by which phytoestrogens induce apoptosis have been identified [34]. Genistein and calycosin induce apoptosis by inhibiting nuclear factor-κB and Akt signaling pathways and anti-apoptotic proteins (Bcl-2) and by activating pro-apoptotic (Bax, Bad) pathways [31,32]. Western blot analysis was used to investigate the effects of aloe emodin, rhapontigenin, and chrysophanol 1-*O*-β-d-glucopyranoside on the apoptotic cell death pathway in MCF-7 cells. Aloe emodin and rhapontigenin induced mitochondria-independent apoptosis by activating the caspase-8 pathway, whereas the cytotoxic effect of chrysophanol 1-*O*-β-d-glucopyranoside was mediated by mitochondria-dependent apoptosis. The distinction between these two apoptotic pathways was estimated by evaluating the presence of two markers, cleaved BID and caspase-8, which are produced only through the mitochondrial pathway. Therefore, *R. undulatum* components (aloe emodin, rhapontigenin, and chrysophanol 1-*O*-β-d-glucopyranoside) may be promising candidates for hormone replacement therapy and chemoprevention of breast cancer because of their estrogenic and breast cancer inhibitory activities.

## 4. Materials and Methods

### 4.1. Chemicals

The Ez-Cytox cell viability assay kit was obtained from the Daeil Lab Service Co. (Seoul, Korea). RIPA buffer, primary antibodies against BH3-interacting domain (BID), Bax, Bcl-2, cleaved caspase-8, cleaved caspase-3, cleaved caspase-9, glyceraldehyde 3-phosphate dehydrogenase (GAPDH), poly ADP ribose polymerase (PARP), and horseradish peroxidase (HRP)-conjugated anti-rabbit secondary antibodies were obtained from Cell Signaling (Danvers, MA, USA). The Pierce™ BCA Protein Assay Kit was obtained from Thermo Scientific (Waltham, MA, USA). RPMI1640 medium was purchased from Cellgro (Manassas, VA, USA). Fetal bovine serum (FBS) and phenol-red-free RPMI medium were obtained from Gibco BRL (Grand Island, NY, USA). Charcoal-dextran-stripped human serum was purchased from Innovative Research (Novi, MI, USA). ECL Advance Western blotting detection reagents were obtained from GE Healthcare (Little Chalfont, UK).

### 4.2. Plant Material

The dried rhizomes of *R. undulatum* were obtained from the Kyung-dong herbal market (Seoul, Korea) and authenticated by Dr. Rack-Seon Seong (Jeonnam Bioindustry Foundation). A voucher specimen (RU201506) was deposited at the Herbarium of College of Pharmacy, Yonsei Institute of Pharmaceutical Sciences, Yonsei University (Incheon, Korea).

### 4.3. Extraction and Isolation

To prepare plant extract samples for in vitro assays, the rhizomes of *R. undulatum* (10 g) were used for each Soxhlet extraction process. For EX1, the sample was extracted with 100% ethyl alcohol (EtOH) (100 mL) using a Soxhlet extractor at 60 °C for 24 h to yield 4.96 g extract. *R. undulatum* was extracted with 50% EtOH (100 mL) at 100 °C for 24 h to yield 5.65 g extract for EX2. For EX3, 10 g of sample was extracted with 50% EtOH (100 mL) at 60 °C for 13 h to obtain 5.85 g extract.

To isolate the active compounds, the rhizomes of *R. undulatum* (6.0 kg) were extracted with MeOH by sonication at 30 °C for 4 h to yield about 700.0 g extract. MeOH is a commonly used solvent for the separation of various materials. The samples then were suspended in H_2_O and successively partitioned with chloroform (CHCl_3_) and ethyl acetate (EtOAc) to obtain CHCl_3_ (RU1, 7.7 g), EtOAc (RU2, 118.0 g), and H_2_O (RU3, 335.0 g) fractions after removing the solvents in a vacuum. 

The EtOAc fraction was subjected to silica gel column chromatography and eluted over a gradient of CHCl3/MeOH (5:1 → 1:1, *v*/*v*), which gave three sub-fractions, RU-2A, RU-2B, and RU-2C. The RU-2A fraction was applied to a silica gel column and eluted with *n*-Hex:EtOAc (2.5:1, *v*/*v*) to give compounds 1 (17 mg) and 3 (14 mg). The RU2B fraction was applied to a silica gel column and eluted with CHCl_3_/MeOH/H_2_O (3.5:1:0.15, *v*/*v*/*v*) to give three smaller fractions, RU2-B1, RU2-B2, and RU2-B3. The RU2-B3 fraction was then applied to a YMC RP-18 column and eluted with MeOH/H_2_O (1:1.2, *v*/*v*) to yield compounds 2 (45 mg) and 4 (100 mg). 

### 4.4. Chromatographic Conditions

To analyze the constituents, the *R. undulatum* extract was filtered and evaporated in vacuo, and then re-suspended in methanol following a previously reported method [35]. This sample solution was filtered through a 0.45 µm membrane filter and analyzed by high-performance liquid chromatography (HPLC). The HPLC system consisted of an Agilent 1290 Infinity liquid chromatography system equipped with a UV-Vis photodiode array detector G4212A and G4220A Quad pump solvent delivery system (Agilent Technologies, Inc., Santa Clara, CA, USA). The output signal of the detector was recorded using an Agilent ChemStation. Chromatographic separation was achieved on a YMC Hydrosphere C18 column (4.6 × 250 mm, 5 µm). Each extract was standardized on the basis of rhaponticin, using HPLC with an acetonitrile–0.1% formic acid gradient over a period of 30 min: acetonitrile 10–90%, 0–30 min at a flow rate of 1.0 mL/min, and monitored at 254 nm. 

### 4.5. Cell Culture

The ER-positive MCF-7 and the ER-negative MDA-MB-231 human breast cancer cell lines were obtained from American Type Culture Collection (ATCC, Manassas, VA, USA). The cell lines were grown in RPMI1640 medium supplemented with 10% FBS, 100 μg/mL streptomycin, and 100 U/mL penicillin, and incubated at 37 °C under a humidified atmosphere with 95% air and 5% CO_2_.

### 4.6. Cell Viability Assay

Cell viability was measured using the Ez-Cytox cell viability detection kit [36]. The cells were incubated in 96-well plates for cell culture at a concentration of 10,000 cells per well for 24 h and treated with the indicated concentrations of the test materials for 24 h. For the antagonistic test, the ER antagonist fulvestrant was added with the test materials. Next, Ez-Cytox reagents were added to each well, and optical density at 450 nm was measured using a microplate reader (PowerWave XS; Bio-Tek Instruments, Winooski, VT, USA) after 1 h to estimate cell viability.

### 4.7. E-Screen Assay

MCF-7 cells were incubated in 24-well plates for cell culture at a concentration of 20,000 cells per well in RPMI1640 medium supplemented with 10% FBS, 100 μg/mL streptomycin, and 100 U/mL penicillin for 24 h. They were then treated with the indicated concentrations of the test materials in phenol red-free RPMI medium supplemented with 5% charcoal-dextran-stripped human serum for 144 h. For the antagonistic test, the ER antagonist fulvestrant was added with the test materials. Ez-Cytox assay reagent was added, after which the optical density value was determined at 450 nm by using a microplate reader (PowerWave XS; Bio-Tek Instruments) to estimate cell viability.

### 4.8. Western Blotting Analysis

After sample treatments, MCF-7 cells incubated in 6-well plates were collected and lysed with RIPA buffer containing 1 mM phenylmethylsulfonyl fluoride on ice. The amounts of each protein were determined using the Pierce™ BCA Protein Assay Kit. Equal amounts of proteins (20 μg/lane) were separated by electrophoresis in a 10% sodium dodecyl sulfate-polyacrylamide gel and electrotransferred onto polyvinylidene difluoride membranes [37]. After blocking with 5% skim milk for 1 h, the proteins in the membrane were incubated at 25 °C for 1 h with primary antibodies against BID, Bax, Bcl-2, cleaved caspase-3, cleaved caspase-8, cleaved caspase-9, PARP, and GAPDH. Following incubation with HRP-conjugated anti-rabbit secondary antibodies at room temperature for 1 h, the expressed proteins were reacted using ECL Advance Western blotting detection reagents and visualized with a FUSION Solo Chemiluminescence System (PEQLAB Biotechnologie GmbH, Erlangen, Germany) according to the manufacturer’s instructions.

### 4.9. Statistical Analysis

Data are presented as means ± standard deviation (SD). Statistical significance was determined using Mann–Whitney U test; *p*-values < 0.05 were considered statistically significant. 

## 5. Conclusions

This study shows the estrogenic and antitumor effects of R. *undulatum* extracts on ER-positive MCF-7 and ER-negative MDA MB 231 breast carcinoma cell lines. R. *undulatum* extracts and three isolated compounds (aloe emodin, rhapontigenin, and chrysophanol 1-*O*-β-d-glucopyranoside) increased the proliferation of MCF-7 cells in a concentration-dependent manner. These effects were attenuated by co-treatment with the anti-estrogen compound fulvestrant, reflecting the estrogenic activity of the R. *undulatum* extract and its ingredients, which was mediated via the ER. In addition, three isolated compounds (aloe emodin, rhapontigenin, and chrysophanol 1-*O*-β-d-glucopyranoside) decreased the viability of MCF-7 cells in a concentration-dependent manner. The cytotoxic effects were unaffected by co-treatment with anti-estrogen compound fulvestrant in MCF-7 cells. Therefore, these cytotoxic effects were independent of the ER. Aloe emodin and rhapontigenin induced mitochondria-independent apoptosis by activating the caspase-8 pathway, whereas the cytotoxic effect of chrysophanol 1-*O*-β-d-glucopyranoside was mediated by mitochondria-dependent apoptosis. Therefore, R. *undulatum* components (aloe emodin, rhapontigenin, and chrysophanol 1-*O*-β-d-glucopyranoside) may be promising candidates for hormone replacement therapy and chemoprevention of breast cancer because of their estrogenic and breast cancer inhibitory activities.

## References

[1] Hoga L., Rodolpho J., Gonçalves B., Quirino B. (2015). Women’s experience of menopause: A systematic review of qualitative evidence. JBI Database Syst. Rev. Implement. Rep..

[2] Bachmann G. (2018). Menopausal urogenital changes: Welcome expansion of management options over the past 25 years. Menopause.

[3] Lobo R.A., Clarkson T.B. (2011). Different mechanisms for benefit and risk of coronary heart disease and stroke in early postmenopausal wosmen: A hypothetical explanation. Menopause.

[4] Danby F.W. (2005). Management of menopause-related symptoms. Ann. Intern. Med..

[5] Anelli A., Gimenez D.L., Rocha A.P., de Abreu C.M., Freitas H.C. (2003). Hormone replacement therapy and the risk of breast cancer: Assessment of therapy acceptance in a cohort of previously treated breast cancer patients. Rev. Hosp. Clin. Fac. Med. Sao Paulo.

[6] Schairer C., Gail M., Byrne C., Rosenberg P.S., Sturgeon S.R., Brinton L.A., Hoover R.N. (1999). Estrogen replacement therapy and breast cancer survival in a large screening study. J. Natl. Cancer Inst..

[7] Willis D.B., Calle E.E., Miracle-McMahill H.L., Heath C.W. (1996). Estrogen replacement therapy and risk of fatal breast cancer in a prospective cohort of postmenopausal women in the United States. Cancer Causes Control.

[8] Dai J., Jian J., Bosland M., Frenkel K., Bernhardt G., Huang X. (2008). Roles of hormone replacement therapy and iron in proliferation of breast epithelial cells with different estrogen and progesterone receptor status. Breast.

[9] Franke H.R., Kole S., Ciftci Z., Haanen C., Vermes I. (2003). In vitro effects of estradiol, dydrogesterone, tamoxifen and cyclophosphamide on proliferation vs. death in human breast cancer cells. Cancer Lett..

[10] Ko S.K., Lee S.M., Whang W.K. (1999). Anti-platelet aggregation activity of stilbene derivatives from *Rheum undulatum*.. Arch. Pharm. Res..

[11] Xiao P., He L., Wang L. (1984). Ethnopharmacologic study of chinese rhubarb. J. Ethnopharmacol..

[12] Kashiwada Y., Nonaka G., Nishioka I. (1984). Studies on Rhubarb (*Rhei Rhizoma)*. VI: Isolation and characterization of stilbenes. ‎Chem. Pharm. Bull..

[13] Lee S.W., Hwang B.S., Kim M.H., Park C.S., Lee W.S., Oh H.M., Rho M.C. (2012). Inhibition of LFA-1/ICAM-1-mediated cell adhesion by stilbene derivatives from *Rheum undulatum*.. Arch. Pharm. Res..

[14] Ye M., Han J., Chen H., Zheng J., Guo D. (2007). Analysis of phenolic compounds in Rhubarbs using liquid chromatography coupled with electrospray ionization mass spectrometry. J. Am. Soc. Mass Spectrom..

[15] Kim I.G., Kang S.C., Kim K.C., Choung E.S., Zee O.P. (2008). Screening of estrogenic and antiestrogenic activities from medicinal plants. Environ. Toxicol. Pharmacol..

[16] Matsuda H., Shimoda H., Morikawa T., Yoshikawa M. (2001). Phytoestrogens from the roots of *Polygonum cuspidatum* (Polygonaceae): Structure-requirement of hydroxyanthraquinones for estrogenic activity. Bioorg. Med. Chem. Lett..

[17] Coopoosamy R., Magwa M. (2006). Antibacterial activity of aloe emodin and aloin A isolated from *Aloe excelsa*.. Afr. J. Biotechnol..

[18] Lee H.S., Lee B.W., Kim M.R., Jun J.G. (2010). Syntheses of resveratrol and its hydroxylated derivatives as radical scavenger and tyrosinase inhibitor. Bull. Korean Chem. Soc..

[19] Kubo I., Murai Y., Soediro I., Soetarno S., Sastrodihardjo S. (1992). Cytotoxic anthraquinones from *Rheum pulmatum*.. Phytochemistry.

[20] Bae K. (2000). The Medicinal Plants of Korea.

[21] Villalobos M., Olea N., Brotons J.A., Olea-Serrano M.F., Ruiz de Almodovar J.M., Pedraza V. (1995). The E-screen assay: A comparison of different MCF7 cell stocks. Environ. Health Perspect..

[22] Fang H., Tong W., Perkins R., Soto A.M., Prechtl N.V., Sheehan D.M. (2000). Quantitative comparisons of in vitro assays for estrogenic activities. Environ. Health Perspect..

[23] Soto A.M., Sonnenschein C., Chung K.L., Fernandez M.F., Olea N., Serrano F.O. (1995). The E-SCREEN assay as a tool to identify estrogens: An update on estrogenic environmental pollutants. Environ. Health Perspect..

[24] Rasmussen T.H., Nielsen J.B. (2002). Critical parameters in the MCF-7 cell proliferation bioassay (E-Screen). Biomarkers.

[25] Zingue S., Nde C.B.M., Michel T., Ndinteh D.T., Tchatchou J., Adamou M., Fernandez X., Fohouo F.T., Clyne C., Njamen D. (2017). Ethanol-extracted Cameroonian propolis exerts estrogenic effects and alleviates hot flushes in ovariectomized Wistar rats. BMC Complement. Altern Med..

[26] Holliday D.L., Speirs V. (2017). *Lannea acida* A. rich. (*Anacardiaceae*) ethanol extract exhibits estrogenic effects and prevents bone loss in an ovariectomized rat model of osteoporosis. Evid. Based Complement. Alternat. Med..

[27] Tung N., Wang Y., Collins L.C., Kaplan J., Li H., Gelman R., Comander A.H., Gallagher B., Fetten K., Krag K. (2010). Estrogen receptor positive breast cancers in BRCA1 mutation carriers: Clinical risk factors and pathologic features. Breast Cancer Res..

[28] Leung E., Kim J.E., Askarian-Amiri M., Finlay G.J., Baguley B.C. (2014). Evidence for the existence of triple-negative variants in the MCF-7 breast cancer cell population. BioMed Res. Int..

[29] Satih S., Chalabi N., Rabiau N., Bosviel R., Fontana L., Bignon Y.J., Bernard-Gallon D.J. (2010). Gene Expression Profiling of Breast Cancer Cell Lines in Response to Soy Isoflavones Using a Pangenomic Microarray Approach. OMICS.

[30] Yang S.H., Zhou Q., Yang X.H. (2007). Caspase-3 status is a determinant of the differential responses to genistein between MDA-MB-231 and MCF-7 breast cancer cells. BBA Mol. Cell Res..

[31] Lee J.Y., Kim H.S., Song Y.S. (2012). Genistein as a potential anticancer agent against ovarian cancer. J. Tradit. Complement. Med..

[32] Chen J., Xiong W.B., Xiong Y., Wu Y.Y., Chen X.J., Shao Z.J., Liu L.T., Kuang W.J., Tan X.S., Zhou L.M. (2011). Calycosin stimulates proliferation of estrogen receptor-positive human breast cancer cells through downregulation of Bax gene expression and upregulation of Bcl-2 gene expression at low concentrations. JPEN J. Parenter. Enter..

[33] Uifălean A., Schneider S., Ionescu C., Lalk M., Iuga C. (2015). Soy Isoflavones and breast cancer cell lines: Molecular mechanisms and future perspectives. Molecules.

[34] Maggiolini M., Bonofiglio D., Marsico S., Panno M.L., Cenni B., Picard D., Ando S. (2001). Estrogen receptor alpha mediates the proliferative but not the cytotoxic dose-dependent effects of two major phytoestrogens on human breast cancer cells. Mol. Pharmacol..

[35] Park S., Kim Y.N., Kwak H.J., Jeong E.J., Kim S.H. (2018). Estrogenic activity of constituents from the rhizomes of *Rheum undulatum* Linné. Bioorg. Med. Chem. Lett..

[36] Lee H.L., Kang K.S. (2017). Protective effect of ginsenoside Rh3 against anticancer drug-induced apoptosis in LLC-PK1 kidney cells. J. Ginseng Res..

[37] Jeon J.H., Kim D.K., Shin Y., Kim H.Y., Song B., Lee E.Y., Kim J.K., You H.J., Cheong H., Shin D.H. (2016). Migration and invasion of drug-resistant lung adenocarcinoma cells are dependent on mitochondrial activity. Exp. Mol. Med..

